# Supplementary material for: Towards Perfect Ultra-Broadband Absorbers, Ultra-Narrow Waveguides, and Ultra-Small Cavities at Optical Frequencies
Source: Nanomaterials (Basel). 2022 Jun 21;12(13):2132. doi: 10.3390/nano12132132 (PMC9268687; doi:10.3390/nano12132132)
Supplement: Supplementary file 1 [file nanomaterials-12-02132-s001.zip › nanomaterials-1763142-supplementary.pdf]

Supplementary Materials: Towards Perfect Ultra-Broadband Absorbers, Ultra-Narrow Waveguides, and Ultra-Small Cavities at Optical Frequencies

Kiyanoush Goudarzi * and Moonjoo Lee*

1. Skin Depth

The skin depth for metals is defined as $\delta = \frac{1}{\mathrm{Im}(k)}$, where $\mathrm{Im}(k) = \frac{\omega}{c\sqrt{2}}\sqrt{\sqrt{\mathrm{Re}(\varepsilon_r)^2 + \mathrm{Im}(\varepsilon_r)^2} - \mathrm{Re}(\varepsilon_r)^2}$. Based on the real and imaginary permittivities of silver and gold materials, the skin depth is calculated at $0.2 < \lambda < 0.6$ μm, as shown in Figure S1.

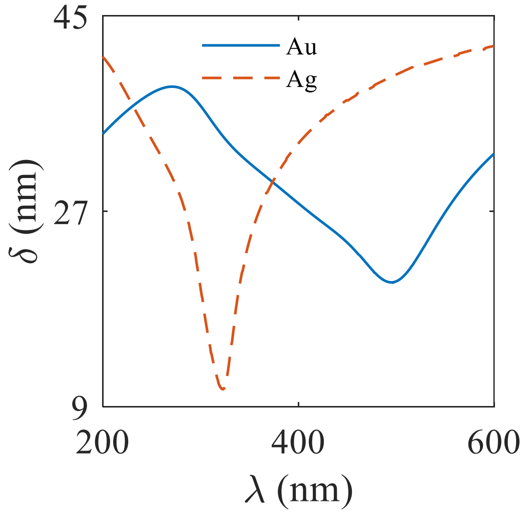

Figure S1. Skin depth for gold and silver over a wavelength range of $0.2 - 0.6$ μm.

2. Electric Hotspots

The normalized electric field distribution over the 2D MPHCs containing silver rods with r = 50 nm and a = 160 nm under the illumination of the TE (Figure S2(a)) and TM (Figure S2(b)) polarized light waves is shown in Figure S2; **P** stands for electric dipoles. Near the surface of the rods, isolated free electrons (either positive or negative) are observed under the TE polarized incident wave that contribute to high-field enhancement near the surface of the rods, creating electric hotspots (Figure S2(a)). In contrast to the TE, no high-field enhancement near the surface under the TM polarized incident light wave appears. As a result, for plasmonic structures, the field enhancement for the 2D MPHCs is revealed under the TE polarized incident wave.

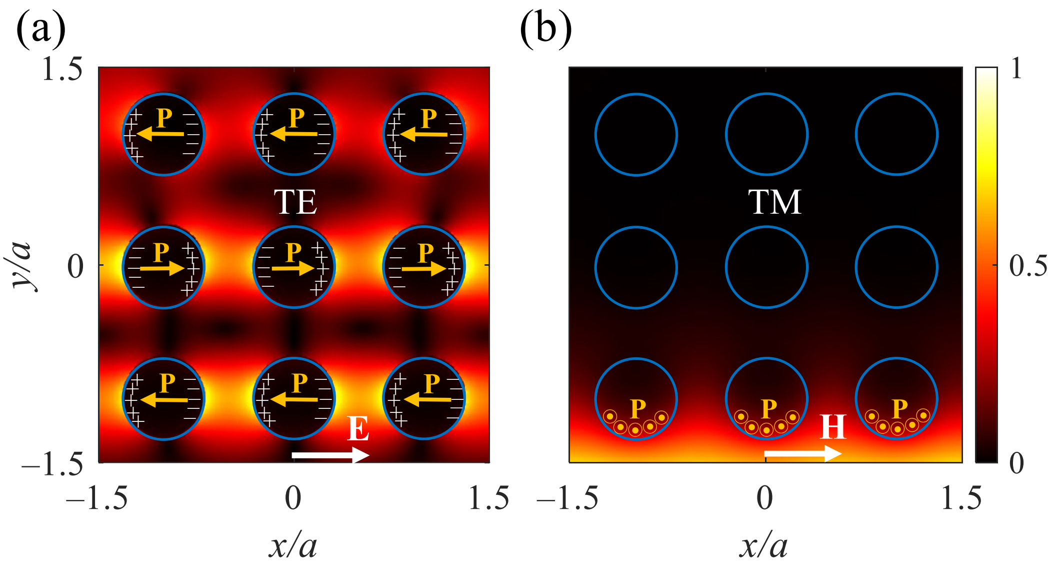

Figure S2. Normalized electrical field distribution over the structure under (a) TE and (b) TM polarized incident plane waves with no position and radius disorderings.

3. Localized Modes in Waveguides

The transmission (T) spectra for the 2D MPHCs consist of 15 × 15 silver rods in air under the TM polarized Gaussian wave for the A-, B-, and C-types of the straight and L-shaped waveguides show a localized mode over $0.4 < \lambda < 0.6$ μm with a maximum amplitude of $T = 0.8$. Approximately 20% of the light is absorbed owing to the intrinsic imaginary permittivity of the silver. There are a few oscillations on the localized guided mode due to the interference of the rods. The transmission spectra remain the same for A and B waveguides but decrease for the C-type. The decrease in the C-type is due to the smaller number of silver rows than that in the other types (Figure S3).

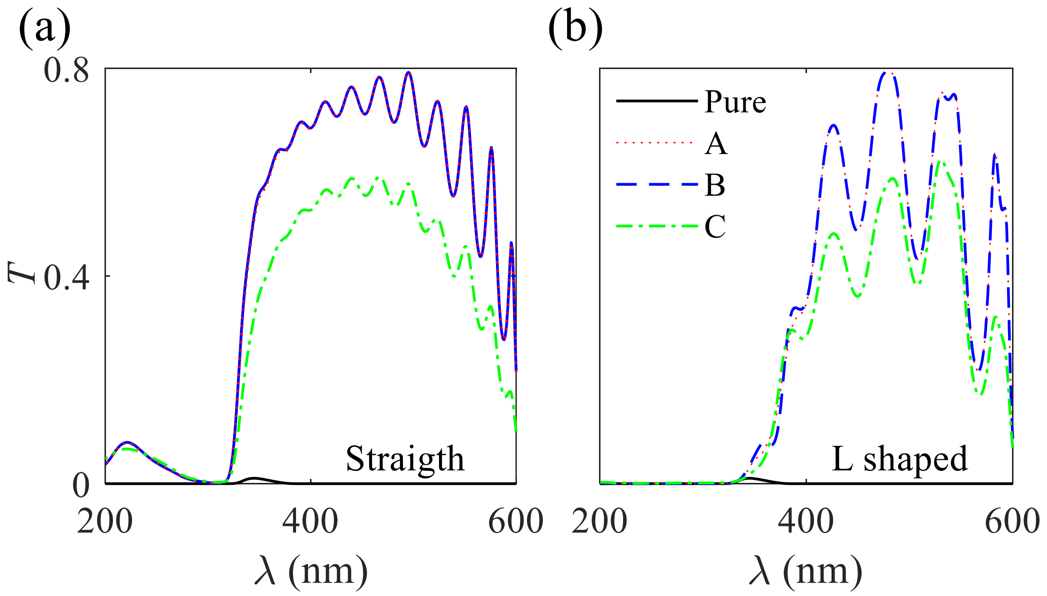

Figure S3. Transmission for 2D MPHCs consists of 15 × 15 silver rods in air under TM polarized Gaussian wave for the A, B, and C types of (a) straight and (b) L-shaped waveguides. For calculating the transmission spectra, no position and radius disorderings are considered.

4. Localized Modes in Cavities

The normalized electric field amplitude for A-, B-, and C-types of the 2D MPHC cavities under the TM polarized dipole sources located at the center of the cavities under

no position and radius disorderings reveal sharp modes with the quality factor of 83, 79, and 27, respectively (Figure S4). The quality factor for the A, B, and C cavities decreases owing to the decreasing circle ring of the silver rods. Reducing the number of rods enables localized electromagnetic waves to escape in the cavity.

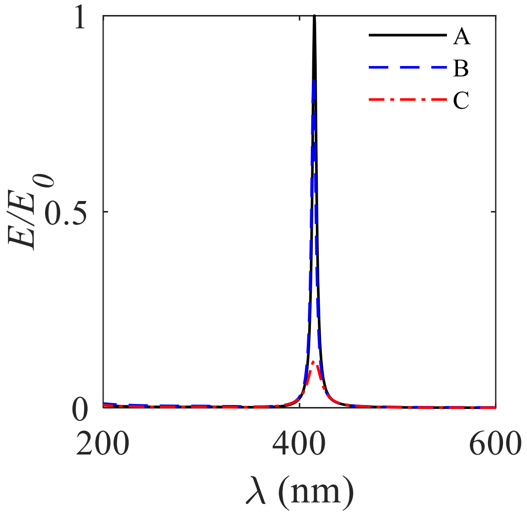

Figure S4. Normalized electric field spectra of 2D MPHCs contain silver rods in air for A, B, and C cavities.
